# Big data–driven personal protective equipment stockpiling framework under a universal healthcare system for disease control and prevention in the COVID-19 era

**DOI:** 10.1017/ice.2020.387

**Published:** 2021-08-03

**Authors:** Kevin Sheng-Kai Ma, Alice Shin-Yi Tsai

**Affiliations:** Department of Health Policy and Management, Johns Hopkins University Bloomberg School of Public Health, Baltimore, Maryland

*To the Editor—*We appreciate the letter by Wang et al^1^ regarding the issue of face masks in protecting against the coronavirus disease 2019 (COVID-19) outbreak. In this letter, we report the distribution of surgical masks on a real-time basis and recognition of the mask holders in need. We hope that by introducing this system to increase the distribution channels, the burden on healthcare providers can be reduced and that the user-friendly interface for PPE providers and consumers may help expedite PPE distribution in a more efficient manner.

Big data analytics has improved healthcare by analyzing electronic medical records, sociodemographic information, and environmental factors.^2^ Moreover, its tracking roles in emerging infectious diseases, including the coronavirus pandemic, have been discussed.^3^ In countries with single-payer universal healthcare systems (UHSs), claimed data of payers could be an abundant source for analytics. On the other hand, compulsory social distancing, coupled with mass masking, has been widely adopted as a strategy for nonspecific symptoms in early-stage COVID-19.^4^ We propose that analytics based on proper concatenation of databases may prevent supply shortages of personal protective equipment (PPE).

Taking Taiwan as an example, cloud-computing–based healthcare databases within the UHS has alleviated the integration between primary care providers and hospitals and has reduced the cost of tracking. Applying the same logistics to PPE allocation would allow PPE providers to manage the distribution of surgical masks on a real-time basis and to recognize the mask holders according to insurance or passport number.^5^ With the help of data analysis, combining artificial intelligence and cloud technology, public health policy making could be practicable. Thus, when it comes to the implementation cost of epidemic prevention policies, Taiwan authorities adopt low-cost, stringent-level strategies compared with other high-income countries, but they still achieved epidemic control in the early outbreak.^6^

After the 2003 severe acute respiratory syndrome (SARS) outbreak, the Taiwan CDC (TCDC) started transferring registered real-time infectious disease data to this established monitoring system, in which PPE stockpiling platform was used. Therefore, prior to the official recognition of COVID-19 outbreak,^3^ PPE databases were subsequently concatenated by UHS to manage resource allocation and logistics when several cases were identified. Establishment of this application programming interface for mask-selling pharmacies under UHS required data transfers as well as managerial issues including governance and ownership, for which interdepartmental communication was efficient within the UHS. Specifically, the tracking system expanded the healthcare informatics system that pharmacists were familiar with, and its user-friendly interfaces for PPE providers and consumers helped expedite distribution processes.^5^ The UHS and the TCDC have also promoted the system to increase the distribution channels, within which government offices may also allot masks to lessen the burden on healthcare providers.

Because masks alone are not effective without combining infection-control measures,^7^ we recommend this integrative platform for the maintenance of more PPE stockpiles, including critical infection-control equipment to reduce iatrogenic SARS-CoV-2 exposure.
